# Habitat heterogeneity drives microbial community assembly and functional specialization in extremely arid ecosystems

**DOI:** 10.1128/aem.02588-25

**Published:** 2026-01-27

**Authors:** Jianrong Huang, Min Cai, Mingxian Han, Baozhu Fang, Lei Dong, Gaosen Zhang, Jia-Rui Han, Shuai Li, Nigora Rustamova, Yonghong Liu, Wen-Jun Li, Hongchen Jiang

**Affiliations:** 1 School of Life Sciences, Henan University 12411, Kaifeng, China; 2 State Key Laboratory of Geomicrobiology and Environmental Changes, China University of Geosciences 12564, Wuhan, China; 3 State Key Laboratory of Ecological Safety and Sustainable Development in Arid Lands, Xinjiang Institute of Ecology and Geography, Chinese Academy of Sciences, Urumqi, China; 4 School of Life Sciences, Sun Yat-Sen University 26469 , Guangzhou, China; 5 Key Laboratory of Extreme Environmental Microbial Resources and Engineering, Northwest Institute of Eco-Environment and Resources, Chinese Academy of Sciences 53045 , Lanzhou, China; 6 Institute of Microbiology, Academy of Sciences of the Republic of Uzbekistan 187919, Tashkent, Uzbekistan; Colorado School of Mines, Golden, Colorado, USA

**Keywords:** microbial ecology, arid environments, habitat heterogeneity, assembly processes, cultivation strategies

## Abstract

Extreme arid ecosystems present significant environmental challenges, yet the mechanisms by which habitat heterogeneity (e.g., salinity gradients, soil–sediment contrasts) shapes microbial community assembly and functional specialization remain poorly understood. This study integrated culture-dependent and culture-independent approaches to investigate microbial diversity, assembly processes, and metabolic potential across wasteland soils, desert soils, and saline lake sediments in the Turpan–Hami Basin. High-throughput sequencing revealed habitat-specific patterns, with lake sediments exhibiting significantly greater OTU richness and Shannon diversity than wasteland and desert soils (*P* < 0.05). These shifts were driven by salinity-dependent taxonomic succession, notably the dominance of *Gammaproteobacteria*, *Halobacteria,* and *Desulfobacteria* in hypersaline lakes. Ecological assembly processes diverged across habitats, with deterministic processes (heterogeneous/homogeneous selection) dominated in deserts and moderate saline lakes, whereas stochastic processes (dispersal limitation, drift) prevailed in wastelands and hypersaline systems. Metabolic profiling highlighted habitat-specific functional specialization: terrestrial systems were characterized by nitrogen-cycling, while saline lakes displayed partitioned sulfur metabolism (e.g., sulfate respiration in high-salinity sediments). Co-occurrence network analyses revealed greater topological complexity in freshwater lakes than in extreme environments, reflecting contrasting resilience strategies. Cultivation strategies informed by sequencing results recovered 4.02% to 21.76% of the sequence-detected genera, significantly improving access to the uncultured majority. These findings demonstrate that habitat heterogeneity drives microbial community assembly and functional evolution in extremely arid ecosystems, underscoring the value of integrating omics with cultivation to uncover microbial dark matter.

**IMPORTANCE:**

Understanding microbial adaptation in hyperarid environments is crucial for predicting ecosystem responses to extreme stressors. This study provides an integrative framework linking environmental heterogeneity to microbial community assembly and metabolic specialization across diverse habitats in one of Earth’s driest basins. Our findings demonstrate that deterministic environmental filtering dominates community assembly in deserts and moderately saline lakes, whereas stochastic processes prevail in wastelands and hypersaline systems. Habitat‑specific metabolic specialization is evident, with nitrogen cycling being key in terrestrial soils and sulfur metabolism central to saline lakes. By significantly improving the recovery of uncultured diversity through targeted strategies, this study bridges a major gap between molecular surveys and cultivable microorganisms. These findings advance ecological theory on community assembly and offer a model for studying microbial resilience and functional evolution under extreme aridity.

## INTRODUCTION

Extreme arid environments, characterized by consistently low precipitation, high temperature fluctuations, and frequent salinity gradients, pose significant challenges to life (1). Despite these harsh conditions, they harbor diverse microbial communities that drive essential biogeochemical cycles, such as carbon, nitrogen, and sulfur transformations, thereby maintaining ecosystem functioning (2–4). Microorganisms in these regions have evolved distinctive adaptive strategies, including desiccation tolerance, osmotic regulation, and metabolic versatility, which not only facilitate survival but also offer potential applications in bioremediation, biotechnology, and astrobiology (5, 6).

However, our understanding of microbial community assembly and function across heterogeneous landscapes in arid regions remains fragmented (7). Although high-throughput sequencing has revealed extensive uncultured microbial diversity in extreme environments (8, 9), key questions persist: How does habitat differentiation, such as lake sediments, wastelands, and deserts, shape microbial diversity and taxonomic composition? To what extent do environmental filters like salinity interact with spatial processes to drive community divergence. Addressing these questions is essential for elucidating the ecological mechanisms underlying microbial resilience in arid ecosystems.

Microbial community structure in extreme environments is governed by a complex interplay of ecological processes, particularly habitat heterogeneity (10–12). Variations in soil and sediment properties, salinity, moisture, and nutrient availability can impose strong environmental filtering, selecting for taxa adapted to local conditions (13, 14). For example, salinity gradients in aquatic systems have been shown to drive distinct microbial taxonomic and functional differentiation, favoring halophilic or halotolerant species in hypersaline environments (15, 16). Microbial community assembly is increasingly viewed as a balance between deterministic selection and stochastic processes (17–19). Deterministic processes, especially environmental filtering, are expected to dominate in harsh habitats where abiotic stressors strongly select for specialized taxa (20, 21). In contrast, stochastic processes such as dispersal limitation and drift may prevail in less extreme or spatially isolated settings (22–24). Elucidating these mechanisms is critical for understanding biodiversity maintenance and ecosystem functioning, particularly in arid systems highly susceptible to disturbance and climate change (25).

Culture-independent techniques, such as 16S rRNA gene sequencing, have revolutionized microbial diversity characterization, revealing unprecedented taxonomic and functional complexity in extreme environments (26, 27). These methods capture the “hidden” majority of microbes that evade traditional cultivation, providing a holistic perspective on community structure and metabolic potential (28, 29). In contrast, culture-dependent methods, though historically limited by a bias toward readily cultivable taxa, remain essential for isolating functional strains, studying their physiology, and unlocking biotechnological applications (30, 31). Integrating these approaches can bridge molecular inventories with tangible biological resources. For instance, metagenomic data can guide targeted cultivation strategies, whereas isolates can help validate predicted metabolic functions (32–34). However, such integrative studies remain scarce in extremely arid regions, where the gap between uncultured and cultured diversity is especially pronounced.

The Turpan-Hami Basin in northwestern China is one of the most extreme arid regions globally, featuring pronounced temperature fluctuations, exceptionally low precipitation, and a diverse mosaic of habitats including wastelands, deserts, and saline/alkaline lakes (35–37). Spanning approximately 256,000 km (2), the basin constitutes 12.59% of Xinjiang’s total land area. Elevations within the basin exhibit a broad gradient, ranging from −124 m to 2,358 m, with a mean elevation of 1,173.36 m (38). This environmental heterogeneity offers an ideal natural system for examining how habitat variation shapes microbial communities. In this study, we systematically characterized microbial community patterns across wasteland soils, desert soils, and lake sediments in the Turpan-Hami Basin. We addressed two key questions: (i) How does habitat heterogeneity affect microbial community assembly mechanisms and functional profiles in these arid ecosystems? (ii) To what extent do culture-independent and culture-based methods yield divergent assessments of microbial diversity, and what biases are associated with each?

To answer these questions, we pursued four key objectives. First, we quantified differences in microbial diversity (alpha and beta diversity) among distinct habitats (wastelands, deserts, and lake sediments) and salinity gradients. Second, we evaluated the relative contributions of deterministic (e.g., environmental filtering) and stochastic (e.g., dispersal limitation and drift) processes in community assembly. Third, we compared metabolic potential and co-occurrence network patterns across habitats. Finally, we assessed the congruence between culturable microbial diversity and the total community captured by sequencing.

## MATERIALS AND METHODS

### Sample collection

Microbial samples were collected systematically from three distinct habitats within the Turpan–Hami Basin, Xinjiang Uygur Autonomous Region (geographical distribution shown in Fig. S1). A total of 266 samples were obtained, comprising 77 wasteland soil samples, 72 desert soil samples (39), and 117 saline lake sediment samples representing four salinity gradients: Chaiwopu Lake (CWP, *n* = 30; salinity of 1.39 g/kg), Dabancheng Lake (DBC, *n* = 10; salinity of 52.8 g/kg), Balikun Lake (BLK, *n* = 54; salinity of 206.80 g/kg), and Huancai Lake (HCH, *n* = 23; salinity of 417.13 g/kg) (37). This sampling strategy encompassed a broad salinity range (1.39–417.13 g/kg), enabling a comprehensive analysis of salinity-dependent microbial community dynamics. All the samples were subsequently divided into three subsamples for physicochemical analysis, microbial cultivation, and microbial community analyses.

### Molecular and bioinformatics analyses

Genomic DNA was extracted from samples collected across different habitats using the FastDNA Spin Kit for Soil (MP Biomedicals, Solon, OH, USA) following the manufacturer’s protocol. For saline lake sediment samples, a sterile stir bar was added to the extraction mixture, which was then stirred continuously at 150 rpm for 16 h at room temperature to enhance cell lysis. The concentration and purity of extracted DNA were determined using a NanoDrop 2000 spectrophotometer (Thermo Fisher Scientific, Wilmington, DE, USA). It should be noted that different primer sets were used for polymerase chain reaction amplification depending on habitat type. For the wasteland and desert soil samples, amplification of the V3-V4 hypervariable region of the 16S rRNA gene was performed via the primer pair 338F (5′-ACT CCT ACG GGA GGC AGC A-3′) and 806R (5′-GGA CTA CHV GGG TWT CTA AT-3′) (39). Sequence libraries were prepared and sequenced on the Illumina NovaSeq 6000 platform (PE250, BioMarker Technology, Beijing). For saline lake sediments, 16S rRNA gene analysis was performed with the universal primers 515F (5′-GTGCCA GCMGCC GCG GTAA-3′) and 806R (5′-GGA CTACHVGGG TWT CTAAT-3′) (40), with library sequencing performed on an Illumina HiSeq2500 system via a paired-end 2 ×250 bp strategy.

Raw 16S rRNA gene sequences were processed in QIIME2 (version 2023.2) (41) following established protocols. The initial processing steps included demultiplexing (*demux-emp-paired*), sequence joining (*vsearch join-pairs*), and quality filtering (*quality-filter q-score-joined* with a *-p-min-quality 20* parameter), with the default parameters except where otherwise specified. Owing to the use of different primer sets, closed-reference operational taxonomic unit (OTU) clustering (97% similarity) was conducted against the SILVA 138 database (*vsearch cluster-features-closed-reference* plugin) (42). Sequences that did not match the reference database were excluded from downstream analyses to maintain consistency across data sets and to enable the subsequent calculation of phylogeny-based metrics (βNTI, βMNTD). Nonbacterial and chloroplast sequences were removed after taxonomic classification, and samples with <5,000 sequences were excluded from downstream analysis. To reduce noise, OTUs represented by fewer than 10 sequences were filtered out. The remaining data were then rarefied to a uniform depth of 5,000 sequences per sample for comparative analysis. The detailed methodological protocols used are described in prior studies (43, 44).

### Microbial community assembly and distance-based redundancy analysis

To elucidate the ecological processes governing microbial community assembly, we employed a null-model approach integrating the beta-nearest taxon index (βNTI) and Raup-Crick metric (RC_bray_) (45, 46). Deterministic processes were inferred when |βNTI| > 2, where βNTI < −2 indicated homogeneous selection and βNTI > 2 reflected variable selection. Stochastic dominance (|βNTI| < 2) was further partitioned via RC_bray_: |RCbray| > 0.95 signifies dispersal limitation (RCbray > 0.95) or homogenizing dispersal (RCbray < −0.95), whereas |RCbray| < 0.95 indicates undominated processes combining weak selection, dispersal, diversification, and drift (47). The calculations were performed via established protocols, with detailed methodologies described in previous studies (44–46). Representative operational taxonomic unit sequences were, therefore, aligned against the SILVA reference alignment. A phylogenetic tree was constructed using a standardized workflow implemented in QIIME2, following established procedures for microbial community phylogenetic inference. The beta mean nearest taxon distance (βMNTD) was calculated via the R function “comdistnt” (abundance.weighted = TRUE; package “picante”). To maintain ecological interpretability, downstream analyses of assembly processes, including the calculation of βNTI and RCbray metrics and the subsequent inference of deterministic versus stochastic contributions, were performed separately for each habitat type (wasteland soils, desert soils, and lake sediments). This habitat-specific analytical approach ensures that the inferred assembly mechanisms reflect the distinct environmental and biotic contexts of each habitat. In addition, to identify the key environmental determinants shaping microbial community structure, distance-based redundancy analysis (dbRDA) based on Bray–Curtis dissimilarity matrices (48) was performed for desert and saline lake habitats only, as physicochemical parameters were not available for the wasteland samples.

### Microbial function prediction and co-occurrence network analysis

To infer the potential metabolic functions of prokaryotic communities, we employed the FAPROTAX pipeline (functional annotation of prokaryotic taxa) (49). This approach is based on mapping taxonomic assignments, which were obtained from the SILVA-based classification of 16S rRNA gene sequences, to the functional groups defined in the FAPROTAX database (version 1.2.6). This database catalogs metabolic and ecological functions for over 4,600 microbial taxa. The resulting OTU table was processed via Python scripts to map the taxonomic assignments to known metabolic functions. We focused on biogeochemical cycles (C, N, S, Fe, and Mn). OTUs with established roles in these cycles were extracted, and their relative abundances were quantified. Importantly, these predictions represent conservative estimates, as FAPROTAX inferences are limited to metabolic capabilities documented in cultured representatives (50, 51).

Microbial ecological networks were constructed to examine co-occurrence patterns among microorganisms via the R package “igraph” (https://igraph.org/) (52, 53), with network visualization performed in Gephi v0.9.7 (http://gephi.github.io/) (54). To reduce network complexity and minimize the influence of rare or sporadic taxa, a two-step filtering process was applied prior to correlation analysis: (1) OTUs with a mean relative abundance <0.01% were excluded and (2) only OTUs present in at least 20% of the samples (occurrence frequency ≥ 1/5) were retained. Statistically significant associations between OTUs were detected through Spearman correlation analysis (implemented via the “WGCNA” package) with stringent thresholds (*r* > 0.75, adjusted *P* < 0.01). In the resulting networks, nodes represented individual OTUs, and edges denoted statistically robust correlations. The threshold values of Zi and Pi were used to evaluate the topological roles of each node (55). The network nodes are divided into four sub-categories, including peripherals (Zi < 2.5, Pi < 0.62), module hubs (Zi > 2.5, Pi < 0.62), connectors (Zi < 2.5, Pi > 0.62), and network hubs (Zi > 2.5, Pi > 0.62) (56). Network topology was characterized by calculating key parameters, including node count, edge number, modularity, closeness centrality, betweenness centrality, eigenvector centrality, diameter, density, clustering coefficient, modularity, robustness, and vulnerability, via “igraph” to provide quantitative measures of network structure and node importance.

### Microbial cultivation from soil and sediment samples

Bacterial strains were isolated from arid soils and saline lake sediments via optimized enrichment protocols tailored to their respective physicochemical properties. For the wasteland and desert soil samples, 5 g of bulk material was transferred to 45 mL of sterile 0.85% (wt/vol) NaCl solution containing 20 sterile 4 mm glass beads. The suspension was vortexed, subjected to ultrasonication (45 kHz, 2 min), and then shaken at 200 rpm and 28°C for 1 h to detach the cells from the particles. Serial dilutions (10^−2^ to 10^−5^) were prepared, with 100 μL aliquots from the 10^−3^ to 10^−5^ dilutions plated onto selective media (Table S1). The choice of media was guided by prior culture-independent analyses, the physicochemical characteristics of the soils, and the recommendations of recent culturomic studies (57), thereby maximizing the recovery of cultivable taxa. All media were autoclaved at 121°C for 15 min, cooled to ≤55°C, and supplemented with trace salts, selective inhibitors, and vitamin B complexes (Table S2). The plates were allowed to air dry in a laminar flow hood for 2–3 days before use. For the saline lake sediments, 5 g samples were homogenized in 45 mL sterile NaCl solution adjusted to the lake’s *in situ* salinity. The suspension was incubated at 37°C with agitation for 50 min to promote cell release and then serially diluted in 0.9% NaCl. One hundred microliter aliquots were plated on the same selective media but with NaCl concentrations ranging from 5% to 25% to match the salinity gradient encountered in the lake. Each dilution series was plated in triplicate. All inoculated plates were incubated at 28°C for 1–4 weeks. Colonies displaying distinct morphologies were repeatedly streaked on fresh agar to obtain pure cultures, which were subsequently cryopreserved in 20% glycerol at −80°C. Genomic DNA was extracted from each isolate, and the 16S rRNA gene was amplified with the universal primers 27F and 1492R following standard protocols (58, 59). PCR products were sequenced, and the resulting near-full-length sequences were compared against the EzBioCloud Database (60) to assign taxonomic identities to the bacterial isolates.

### Statistical analysis

Alpha diversity metrics (species richness and Shannon indices) were calculated using the *pd* and *diversity* functions within the “picante” package (v1.8.2) in R v4.5.1 (61). Microbial community composition differences were visualized via principal coordinate analysis (PCoA) based on Bray‒Curtis dissimilarity matrices implemented in the “microeco” package (62). The statistical significance of compositional differences was assessed using permutational multivariate analysis of variance (PERMANOVA) with the *adonis* function (Bray‒Curtis distance, 999 permutations). For the comparative analysis of microbial communities obtained through culture-independent and culture-dependent approaches, Venn diagrams were generated. Venn diagrams were generated via the “jvenn” tool (https://jvenn.toulouse.inra.fr/app/index.html) (63) to visualize shared and unique taxonomic groups between methodologies.

## RESULTS

### Habitat-specific microbial community diversity and composition

**Fig 1:**
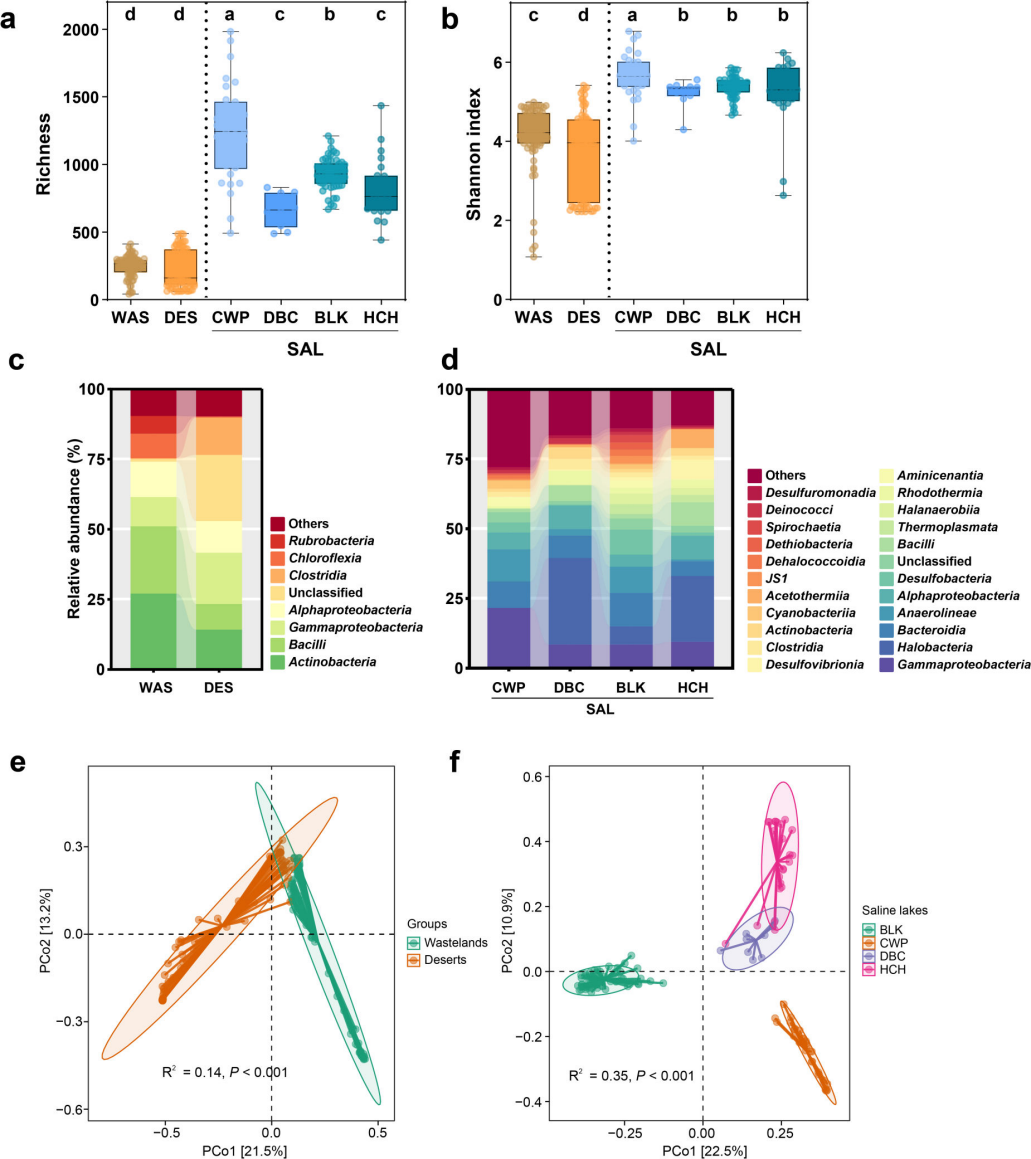
Variation in microbial community diversity and composition across habitats in the Turpan–Hami basin. (**a**) Richness and (**b**) Shannon indices, which represent α diversity metrics. Different lowercase letters above the boxplots denote statistically significant differences (*P* < 0.05, Wilcoxon rank-sum test). (**c, d**) Genus-level community composition: (**c**) contrasting wastelands and deserts, (**d**) elucidating saline lake environments. PCoA based on the Bray‒Curtis dissimilarity matrix, showing the difference in microbial community composition in (**e**) wastelands and deserts and (**f**) saline lakes (Adonis tests, permutations = 999). Abbreviations: BSS, Wastelands; DES, Deserts; SAL, Saline lakes; CWP, Chaiwopu Lake; DBC, Dabancheng Lake; BLK, Balikun Lake; HCH, Huancai Lake.

Alpha diversity analyses revealed significant differences in microbial community structure among the surveyed habitats (Fig. 1). Lake sediments presented significantly greater OTU richness (*P* < 0.05, Fig. 1a) and Shannon diversity (*P* < 0.05, Fig. 1b) than both wasteland and desert soils did. The freshwater lakes presented greater species richness than did the saline lakes (*P* < 0.05), whereas increasing lake salinity was correlated with a significant decline in microbial diversity (*P* < 0.05, Fig. 1b).

The microbial community structure of each habitat was distinct (Fig. 1c). Wasteland soils were dominated by *Actinobacteria* (26.99%), *Bacilli* (23.97%), *Alphaproteobacteria* (12.58%), and *Gammaproteobacteria* (10.44%). The desert soils presented a high proportion of unclassified taxa (23.62%), *Gammaproteobacteria* (18.27%), *Actinobacteria* (14.10%), *Clostridia* (13.28%), and *Alphaproteobacteria* (11.28%). Lake sediment communities differed markedly, with *Gammaproteobacteria*, *Halobacteria*, *Bacteroidia*, *Anaerolineae*, *Alphaproteobacteria*, *Desulfobacteria*, *Bacilli*, and *Thermoplasmata* representing the predominant classes (relative abundance >1%, Fig. 1d). Furthermore, salinity gradients shaped the taxonomic patterns within the lake sediments. The freshwater CWP was dominated by *Gammaproteobacteria* (21.47%), *Anaerolineae* (11.55%), *Bacteroidia* (9.44%), and *Alphaproteobacteria* (6.04%). Moderate-salinity DBC was characterized by *Halobacteria* (31.03%), *Alphaproteobacteria* (8.40%), *Gammaproteobacteria* (8.40%), *Bacteroidia* (8.06%), and *Bacilli* (8.06%). In high-salinity BLK, *Bacteroidia* (11.95%), *Anaerolineae* (9.44%), *Desulfobacteria* (8.97%), *Gammaproteobacteria* (8.37%), and *Halobacteria* (6.56%) were prominent. The hypersaline HCH was strongly dominated by *Halobacteria* (23.59%), *Gammaproteobacteria* (9.38%), *Alphaproteobacteria* (8.52%), *Desulfovibrionia* (7.01%), and *Acetothermiia* (6.67%). This pattern revealed salinity-dependent succession, with increased representation of halophiles (e.g., *Halobacteria*) and sulfate reduction-related taxa (e.g., *Desulfobacteria* and *Desulfovibrionia*) along the salinity gradient. Principal coordinate analysis (PCoA) confirmed these compositional shifts, revealing distinct clustering patterns. Wasteland and desert soil communities formed clearly separate clusters (Fig. 1e), indicating significant compositional differences between these terrestrial ecosystems. Similarly, the lake sediment communities clustered according to their respective salinity levels (Fig. 1f).

### Habitat-specific microbial community assembly processes and their environmental determinants

**Fig 2:**
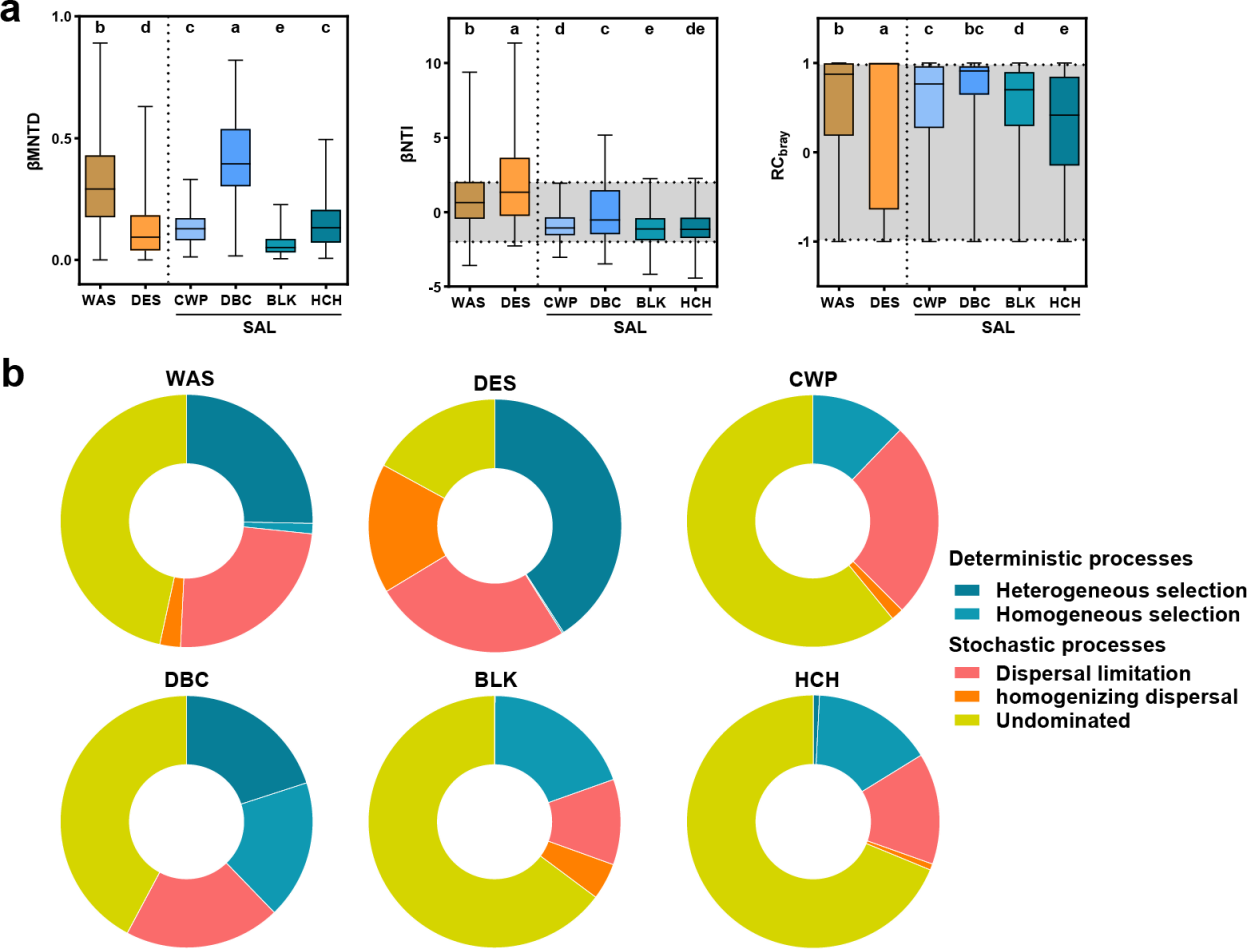
Assembly mechanisms of microbial communities across diverse habitats in the Turpan–Hami basin. (**a**) Quantitative assessments of βMNTD, βNTI, and RC_Bray_ in microbial communities stratified by habitat. Significant intergroup divergences are denoted by different lowercase letters above the box plots (*P* < 0.05, Wilcoxon rank-sum test). (**b**) Synthesis of the ecological processes governing microbial community composition, as inferred through null model analyses.

Analysis of ecological assembly processes revealed significant differences in βMNTD among habitat types (*P* < 0.05), confirming variations in microbial community composition (Fig. 2). Using the iCAMP framework with βNTI and RCbray metrics, the relative contributions of deterministic versus stochastic processes were quantified. Desert soils and moderate-salinity DBC lake sediments presented |βNTI| > 2 for most taxa, indicating that deterministic processes dominated the assembly (Fig. 2a). Deterministic processes contributed substantially to the assembly in desert soils and DBC lakes, respectively (Fig. 2b). Desert soil communities were shaped primarily by heterogeneous selection, whereas DBC lakes presented combined influences of heterogeneous and homogeneous selection. In contrast, stochastic processes were dominant in wasteland soils, CWP, BLK, and HCH habitats, with undominated processes (weak selection, diversification, and drift) prevailing. Dispersal limitation emerged as another key stochastic factor across all habitats (Fig. 2b).

Distance-based redundancy analysis (dbRDA) was employed to identify the key environmental determinants shaping the microbial community structure in desert and saline lake habitats (Fig. S2). dbRDA revealed that salinity and pH explained 52.09% and 23.11% of community variation in desert soils (*P* < 0.01), respectively (Fig. S2a). Microbial community compositions were significantly correlated with aridity, salinity, total organic carbon (TOC), total nitrogen (TN), and nitrate (NO_3_^−^) concentration. In the saline lake habitat, the dbRDA model accounted for a higher cumulative variation (>90%) (Fig. S2b). The ordination revealed a clear separation among samples along environmental gradients. Salinity, pH, and nutrient availability (represented by TOC, TN, NO_3_^−^, NH_4_^+^) were identified as the significant environmental drivers.

### Metabolic potential and co-occurrence network analysis

**Fig 3:**
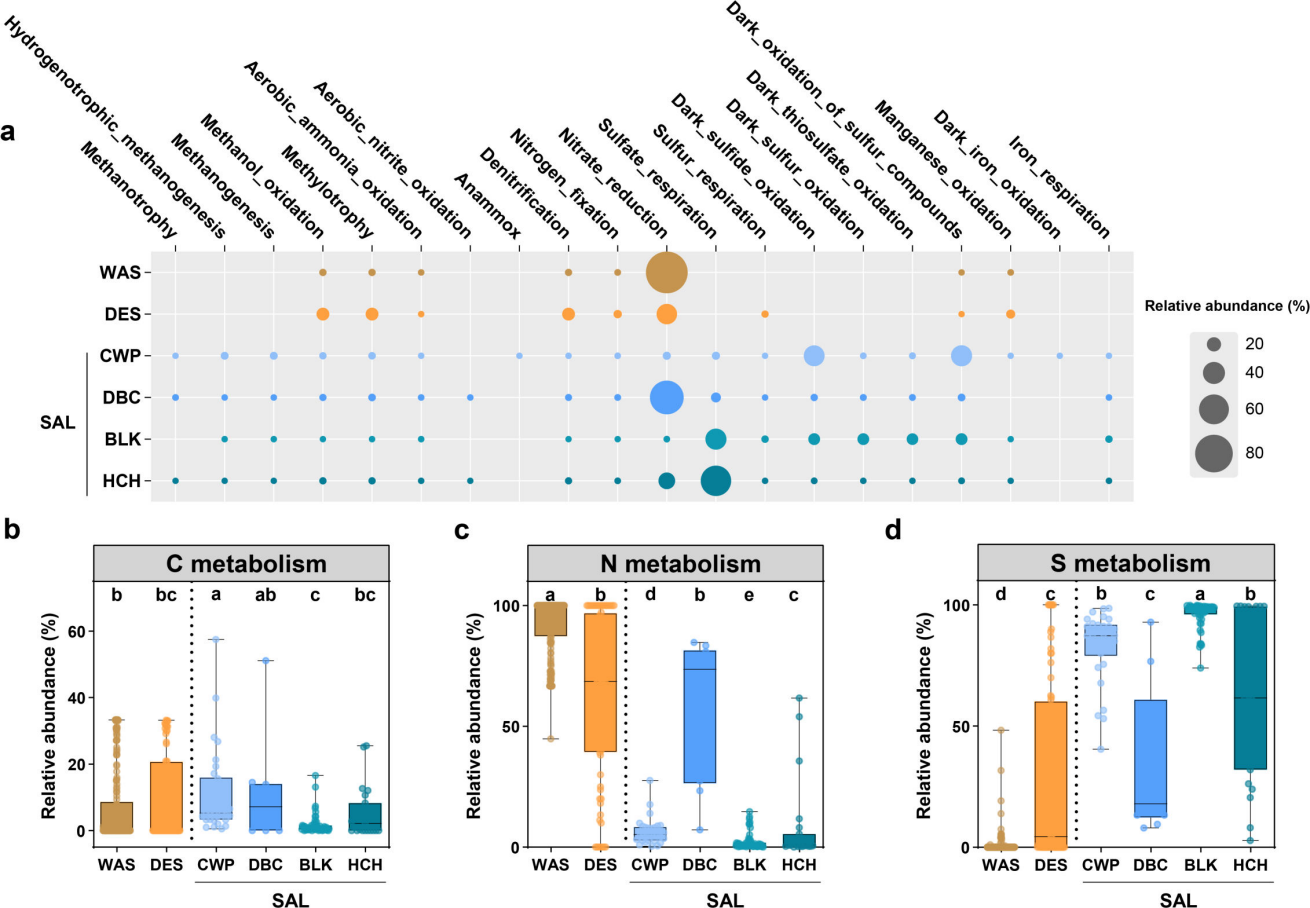
Predicted metabolic pathways of microorganisms across habitats in the Turpan–Hami Basin inferred via FAPROTAX. (**a**) Functional profiles of microbial communities across six regional habitat types, where circle size denotes relative abundance estimates of predicted microbial functions. (**b–d**) Comparative analyses of metabolic pathways: (**b**) C, (**c**) N, and (**d**) S, as quantified by relative abundance, with statistical significance denoted by letters (Wilcoxon test, *P* < 0.05).

FAPROTAX analysis revealed distinct metabolic functional profiles among wasteland soils, desert soils, and lake sediments (Fig. 3). Nitrogen cycling processes were prominent in terrestrial habitats, collectively accounting for 94.17% and 57.80% of the predicted functions in wasteland and desert soils, respectively (Fig. 3c). Wasteland soils specialize in nitrate reduction (90.54%), with minor contributions from methanol oxidation (2.45%) and denitrification (2.25%). The desert soils presented more diverse nitrogen metabolism, including nitrate reduction (36.04%), denitrification (16.49%), and nitrogen fixation (5.05%), as well as substantial methanol oxidation (16.52%) and manganese oxidation (6.85%) (Fig. 3c). In contrast, sulfur metabolism was central to the saline lake sediments, with process partitioning along salinity gradients (Fig. 3d): freshwater CWP specialized in dark sulfide oxidation (37.59%); moderate-salinity DBC combined sulfate respiration (9.05%) with dark sulfide oxidation (3.44%); high-salinity BLK showed diverse sulfur transformations (sulfate respiration [37.60%], dark sulfur/thiosulfate oxidation [13.39%–13.90%]); and hypersaline HCH was dominated by sulfate respiration (61.52%).

**Fig 4:**
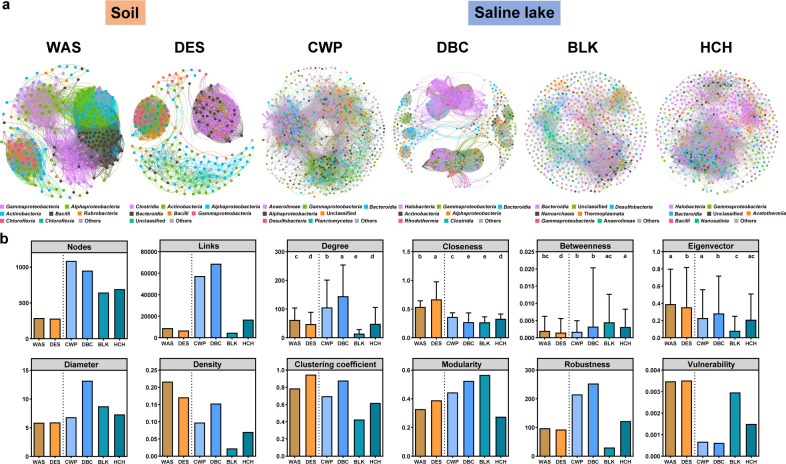
Microbial community co-occurrence networks and topological characteristics across habitats in the Turpan–Hami Basin. (**a**) Cooccurrence network models of microbial communities at the OTU level in six regions; node size is scaled to relative abundance. (**b**) Topological characteristics of the networks, presented as a bar chart. Statistically significant intergroup differences are indicated by distinct lowercase letters above the bars (*P* < 0.05, Wilcoxon rank-sum test).

Co-occurrence network analysis revealed distinct interaction patterns among microbial communities in different habitats (Fig. 4). Compared with wasteland soils (289 nodes, 9,019 edges), desert soils (281 nodes, 6,727 edges), and saline lakes (DBC: 950 nodes, 68,889 edges; BLK: 646 nodes, 4,702 edges; HCH: 692 nodes, 16,859 edges), freshwater lake CWP presented significantly greater network complexity (1,085 nodes and 57,426 edges) (Fig. 4a), suggesting greater microbial interactions in freshwater sediments. Degree distribution analysis revealed significantly greater connectivity in CWP and DBC (*P* < 0.05), indicating tighter microbial cooperation in these habitats. In contrast, soil environments (WAS and DES) presented significantly elevated closeness and Eigenvector centrality (*P* < 0.05) and higher vulnerability, suggesting a strategy that prioritizes efficient information or resource transfer efficiency, albeit at the cost of higher community fragility in low-diversity, extreme terrestrial environments. The CWP and DBC communities also displayed highest levels of network robustness, indicating robust ecological resilience and a superior adaptive capacity to environmental fluctuations.

To further evaluate the ecological roles of individual taxa within microbial networks, nodes were classified into peripherals, module hubs, connectors, and network hubs based on within module and among module connectivity (Fig. S3). The abundance and taxonomic composition of keystone taxa varied markedly across habitats, revealing a strong environmental gradient in network organization. Terrestrial environments exhibited a pronounced scarcity of keystone taxa. The wasteland soil network consisted exclusively of peripheral nodes, indicating the absence of highly connected or structurally influential taxa. Desert soils contained only a small number of module hubs, primarily affiliated with *Alphaproteobacteria*, *Actinobacteria*, and *Acidimicrobiia*, suggesting limited but detectable centralization within modules. In contrast, aquatic habitats displayed substantially more complex topological structures. The freshwater lake CWP harbored multiple module hubs spanning *Anaerolineae*, *Bacteroidia*, and *Gammaproteobacteria*, together with a connector taxon from *Gemmatimonadetes*, reflecting enhanced cross- module integration. With increasing salinity from freshwater to hypersaline conditions, both the diversity and prominence of keystone taxa increased markedly. Network hubs were absent in low salinity and terrestrial habitats but emerged in saline lakes, first appearing in BLK and becoming highly abundant in the hypersaline HCH network. The HCH network was characterized by a high density of network hubs, connectors, and module hubs dominated by halophilic and salt tolerant lineages, including *Halobacteria*, *Bacilli*, *Actinobacteria*, *Bacteroidia*, and *Rhodothermia*. Collectively, these patterns indicate that increasing salinity promotes the emergence of highly connected keystone taxa and drives a transition toward more centralized and hierarchically structured microbial interaction networks under extreme environmental conditions.

### Culturable microbial diversity and predicted functions across Turpan-Hami Basin habitats

**Fig 5:**
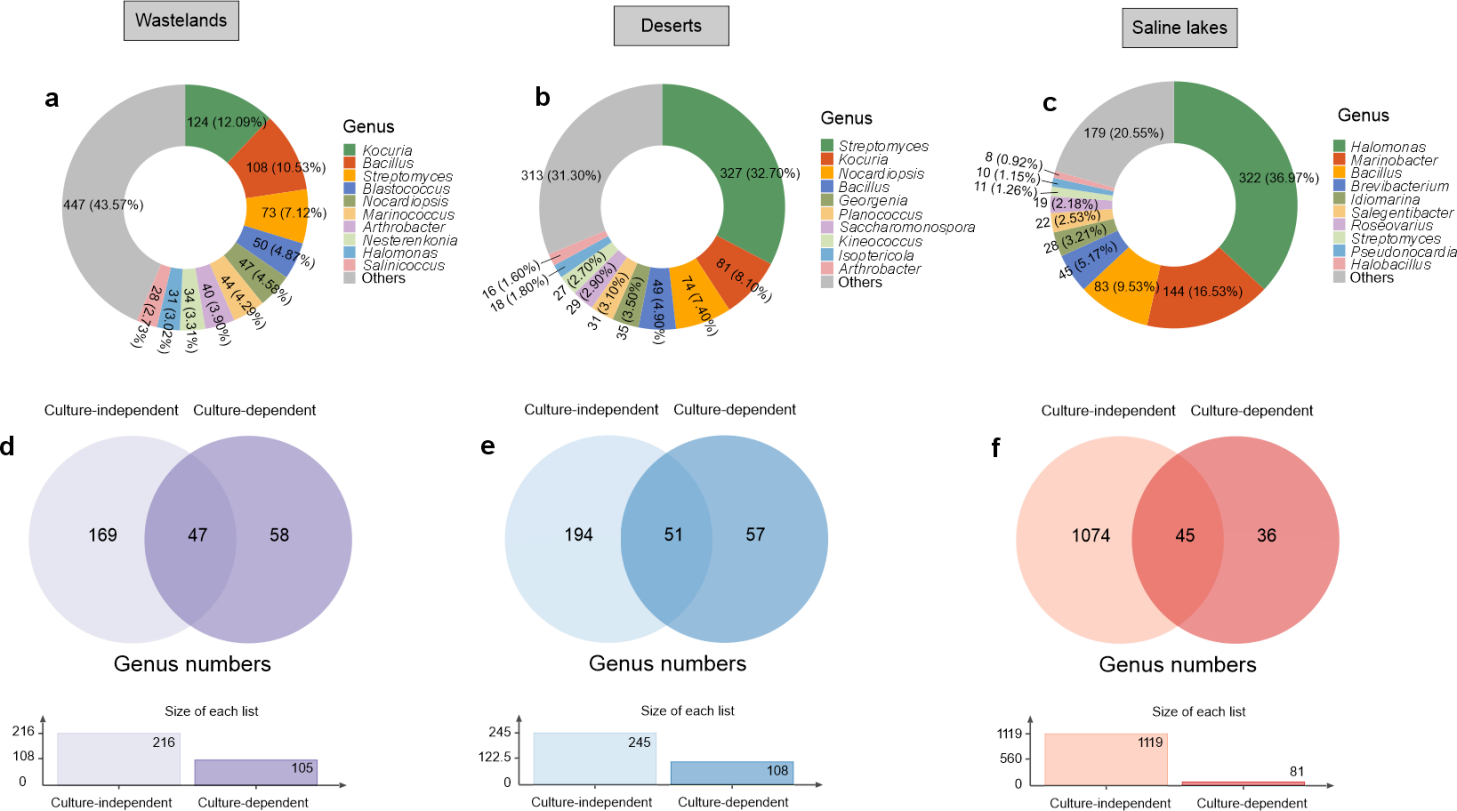
Composition of culturable microbial communities in extremely arid environments of the Turpan-Hami Basin. (**a–c**) Community composition across distinct habitats: (**a**) wastelands, (**b**) deserts, and (**c**) saline lakes. (**d–f**) Venn diagram illustrating overlaps and distinctions in microbial communities revealed by culture-independent and culture-dependent methods in wastelands (**d**), deserts (**e**), and saline lakes (**f**).

A significant improvement in culture-dependent diversity was achieved through isolation and cultivation strategies designed according to culture-independent sequencing findings and intrinsic sample properties. A total of 1,026, 1,000, and 871 bacterial strains were successfully isolated from wasteland soil, desert, and saline lake environments, respectively, via a standardized dilution plating method. The taxonomic composition varied significantly among these habitats, with the most abundant genera exhibiting clear ecological differentiation. In the wasteland soil samples, *Kocuria* (12.09%, 124 strains), *Bacillus* (10.53%, 108 strains), *Streptomyces* (7.12%, 73 strains), *Blastococcus* (4.87%, 50 strains), and *Nocardiopsis* (4.58%, 47 strains) were the most frequently recovered genera, followed by *Marinococcus* (4.29%, 44 strains), *Arthrobacter* (3.90%, 40 strains), *Nesterenkonia* (3.31%, 34 strains), *Halomonas* (3.02%, 31 strains), and *Salinicoccus* (2.73%, 28 strains). The remaining isolates, representing 43.57% (447 strains) of the total, belonged to other, less abundant genera (Fig. 5a). The desert soil samples presented a distinct community structure characterized by a strong dominance of *Streptomyces* (32.70%, 327 strains). *Kocuria* (8.10%, 81 strains), and *Nocardiopsis* (7.40%, 74 strains) were also prevalent, followed by *Bacillus* (4.90%, 49 strains), *Georgenia* (3.50%, 35 strains), *Planococcus* (3.10%, 31 strains), *Saccharomonospora* (2.90%, 29 strains), *Kineococcus* (2.70%, 27 strains), *Isoptericola* (1.80%, 18 strains), and *Arthrobacter* (1.60%, 16 strains). The remaining isolates, accounting for 31.30% (313 strains) of the total, included various other genera (Fig. 5b). In contrast, the saline lake samples presented a distinct microbial community adapted to high-salinity conditions. *Halomonas* was the dominant genus (36.97%, 322 strains), with *Marinobacter* (16.53%, 144 strains) and *Bacillus* (9.53%, 83 strains) also being abundant. The community also included *Brevibacterium* (5.17%, 45 strains), *Idiomarina* (3.21%, 28 strains), *Salegentibacter* (2.53%, 22 strains), *Roseovarius* (2.18%, 19 strains), *Streptomyces* (1.26%, 11 strains), *Pseudonocardia* (1.15%, 10 strains), and *Halobacillus* (0.92%, 8 strains). The remaining isolates, which represented 20.55% (179 strains) of all the isolates, were composed of other varied, less abundant genera (Fig. 5c).

FAPROTAX-based functional prediction revealed clear habitat-specific differentiation in the metabolic potential of culturable microbial isolates across the three habitats (Fig. S4). Isolates from the saline lake habitat showed a pronounced enrichment in carbon metabolism, accounting for more than 20% of the predicted functions. This pattern was largely driven by elevated contributions from hydrocarbon degradation and ligninolysis pathways. In addition, sulfur metabolism-related functions, including sulfur compound oxidation, were uniquely enriched in saline lake isolates, highlighting adaptation to sulfur-rich and redox variable conditions. In contrast, nitrogen metabolism was most prominent in the desert habitat, with nitrate reduction and ureolysis representing the dominant functional categories. This suggests an enhanced capacity for nitrogen transformation under nutrient limited and arid soil conditions. Isolates from wasteland soils displayed a distinct functional signature characterized by a relatively higher abundance of aromatic compound degradation, indicating potential specialization in the turnover of complex organic substrates. These results indicate that the functional potential of the culturable microbial fractions is highly specialized and corresponds to the unique geochemical characteristics of their respective ecological niches.

### Comparative analysis of cultured and uncultured microbial diversity

While conventional cultivation approaches typically recover < 1% of the environmental microbiota, our integrated strategy combining enrichment culturing with metagenomics significantly enhanced microbial isolation across Turpan–Hami Basin habitats. High-throughput sequencing detected 216, 245, and 1,119 genera in wasteland soils, desert soils, and saline lakes, respectively. Cultivation recovered 105, 108, and 81 genera, representing 21.76%, 20.82%, and 4.02%, respectively, of the sequence-detected taxa in each habitat (Fig. 5d through f). This improvement (4 to 20 times) over traditional cultivation yields (typically ~1%) demonstrates the efficacy of targeted enrichment strategies for accessing the “uncultured majority.” Notably, hypersaline lakes presented lower culturability (4.02%) despite greater total diversity, suggesting stronger physiological constraints on isolation. These cultivated collections establish a valuable germplasm resource for discovering novel taxa, genes, and functions from extreme environments.

## DISCUSSION

### Habitat heterogeneity affects microbial community diversity and composition

The observed divergences in microbial diversity and community composition across Turpan-Hami Basin habitats—wasteland, desert, and lake sediments—underscore the profound influence of environmental heterogeneity on microbial community assembly in these arid ecosystems. Lake sediments, particularly freshwater systems, present significantly richer and more diverse microbial communities than terrestrial habitats do (Fig. 1). This disparity likely stems from the relatively stable moisture and nutrient conditions prevalent in the aquatic environments of arid regions, which can support more complex microbial assemblages (64, 65). Furthermore, the freshwater lakes surpassed the saline lakes in terms of species richness (Fig. 1a). This pattern can be attributed to the lower osmotic stress in freshwater systems, enabling the proliferation of taxa less adapted to the constraints imposed by high salinity (66). This observation aligns with prior studies demonstrating that elevated salinity acts as an environmental filter, curtailing niche breadth for osmotically sensitive microorganisms (67, 68). The negative correlation between lake salinity and microbial diversity reinforces salinity as a key determinant of community structure (69).

The terrestrial habitats presented distinct compositional signatures. Wasteland soils are dominated by *Actinobacteria*, a group that is well adapted to desiccation and organic matter degradation (70). Desert soils are characterized by a high proportion of unclassified taxa, complemented by stress-tolerant classes (e.g., *Gammaproteobacteria*, *Actinobacteria*, *Clostridia*) (71). These variations likely reflect differences in soil organic carbon content, moisture availability, and disturbance regimes (e.g., wind erosion) (72). The successional patterns of halophiles (e.g., *Halobacteria*) and sulfate reduction-related taxa (e.g., *Desulfobacteria* and *Desulfovibrionia*) along the salinity gradient reflect functional adaptations to osmotic stress and energetic metabolism strategies in high-salt environments (73). The synthesis of compatible solutes by *Halobacteria*, for example, mitigates osmotic pressure (74). Sulfate-reducing taxa thrive in anoxic, saline conditions by utilizing sulfate as a terminal electron acceptor, playing critical roles in maintaining ecosystem function under extreme salinity stress (75, 76). The PCoA results reinforced habitat-specific clustering, with clear separation between terrestrial ecosystems (wasteland vs desert) and salinity gradients in lake sediments, confirming that habitat type and salinity act as key drivers of community structuring. Overall, these findings emphasize that microbial community diversity and composition in the Turpan-Hami Basin are jointly shaped by habitat type and selective environmental filters, with salinity emerging as a primary determinant within aquatic systems.

### Contrasting ecological processes govern microbial community assembly

Our analysis of community assembly processes revealed that habitat-specific environmental constraints dictate distinct equilibria between deterministic and stochastic processes. Deterministic processes dominated community assembly in desert soils and moderate-salinity lakes (DBC) (Fig. 2). Desert communities are shaped primarily by heterogeneous selection, which is likely driven by intermittent resource availability and extreme temperature fluctuations, which are characteristic of arid soils (77). This interpretation is further supported by the dbRDA results, which identified aridity, salinity, total organic carbon, total nitrogen, and nitrate as significant correlates of microbial community composition in desert habitats (Fig. S2a). Together, these results indicate that multiple abiotic constraints jointly drive deterministic selection in desert soils. In the DBC lake, the combination of heterogeneous and homogeneous selection potentially reflects localized environmental gradients, such as microscale salinity variations, that are superimposed on broader stabilizing pressures (78). The dbRDA analysis revealed that salinity and nutrient-related variables were significantly associated with community differentiation, suggesting that moderate salinity environments impose sufficiently strong but spatially structured filters to promote deterministic assembly. These findings are consistent with theoretical frameworks suggesting that deterministic processes prevail in harsh or resource-limited environments, where strong environmental filters select for adapted taxa (19, 79, 80).

In contrast, stochastic processes (e.g., drift, dispersal limitation) governed assembly in wasteland soils, freshwater lake (CWP), high-salinity lake (BLK), and hypersaline lake (HCH), with undominated processes accounting for more than 46% of the total contributions. Wasteland soils, with more moderate aridity than deserts do, may experience weaker environmental filtering, allowing stochastic colonization and demographic drift to exert greater influences (81). In saline lakes with high and extreme salinity, stochasticity in HCH and BLK may arise from periodic disturbances (e.g., evaporation-driven salinity spikes) or dispersal limitations because extreme conditions restrict microbial movement. Notably, dbRDA results showed that salinity and pH were strongly correlated with microbial community variation in saline lake sediments, indicating that although environmental gradients influence community composition, stochastic processes can still dominate assembly under highly variable or disturbance-prone conditions. Dispersal limitation has emerged as a consistent stochastic factor across all habitats, underscoring the isolation of microbial communities in fragmented arid landscapes (82). Collectively, these results demonstrate that habitat heterogeneity regulates microbial assembly by modulating the relative strength of deterministic environmental filtering and stochastic processes. Deterministic processes dominate where environmental constraints are strong and structured, whereas stochasticity prevails in habitats characterized by weaker, more variable, or intermittently disruptive filters.

### Metabolic specialization and network topology reflect habitat adaptations

FAPROTAX and co-occurrence network analyses revealed significant links between microbial function, community interactions, and habitat characteristics. Terrestrial habitats specialize in nitrogen cycling, with wasteland soils specializing in nitrate reduction, likely as adaptations to episodic nutrient inputs such as aeolian deposition (4). Desert soils expand into denitrification and nitrogen fixation, reflecting strategies to conserve limiting nitrogen under oligotrophic conditions (83). The prevalence of methanol oxidation in deserts further suggests the utilization of volatile organic compounds as alternative carbon sources (84), a common adaptation in nutrient-poor arid systems. In contrast, saline lakes exhibit salinity-dependent specialization in terms of sulfur metabolism (85). The freshwater CWP focused on dark sulfide oxidation, whereas high-salinity BLK and HCH promoted sulfate respiration. This pattern is consistent with the enrichment of sulfate-reducing taxa such as *Desulfobacteria* and *Desulfovibrionia* in hypersaline environments (86). Such metabolic partitioning likely reflects shifts in electron acceptor availability and energetic constraints across salinity gradients. The habitat-specific metabolic patterns inferred from high-throughput sequencing were further corroborated by functional profiling of culturable microbial isolates (Fig. S4). Together, these results indicate that the functional potential of both the total and the culturable microbial fractions is highly specialized and corresponds to the distinct geochemical constraints of each habitat.

Co-occurrence networks mirrored these functional patterns. The freshwater CWP displayed greater complexity, characterized by a greater number of nodes and edges and elevated betweenness centrality (Fig. 4). This finding indicates that relatively robust, interconnected communities buffer against disturbances (87). Terrestrial soils (WAS and DES), in turn, presented elevated closeness and eigenvector centrality, suggesting streamlined resource and information transfer in low-diversity, extreme environments. However, the higher vulnerability and low robustness observed in these soil networks suggest that their simplified structure may be more susceptible to environmental fluctuations (88). As salinity increased, the network topology in saline and hypersaline lakes (BLK and HCH) shifted toward distinct adaptive configurations. In BLK, the network exhibited the highest modularity, reflecting a highly compartmentalized structure. Such high modularity is often interpreted as an ecological strategy to limit the spread of environmental perturbations across the entire community, thereby preserving overall network stability (89). The concentration of interactions within specific modules in BLK may also indicate specialized metabolic niches driven by increasing chemical heterogeneity in saline sediments (90). In the hypersaline HCH, despite the extreme environmental filter of salinity, the network maintained a relatively high edge count (16,859) and was supported by keystone taxa. Unlike the terrestrial and freshwater sites, the HCH network was anchored by a diverse array of network hubs and connectors primarily composed of halophilic and salt-tolerant lineages, such as *Halobacteria*, *Bacilli*, and *Nanosalinia*. The presence of six *Halobacteria* network hubs suggests that these taxa act as “biological glues,” facilitating inter-module communication and maintaining community integrity under intense osmotic stress (91, 92). This shift from a complexity-driven stability in CWP to a keystone-driven stability in HCH indicates that under extreme salinity, the community relies on a core group of specialized taxa to coordinate metabolic cross-feeding and collective survival (93). These findings highlight that microbial networks undergo fundamental structural reorganizations to sustain ecological functions across a spectrum of environmental stress.

### Culturable diversity and the “uncultured majority”: bridging gaps in extreme environments

Our culturomic survey elucidated habitat-specific patterns in culturable bacterial genera, complementing culture-independent data and highlighting the utility of targeted enrichment strategies. Wasteland soils are dominated by *Actinobacteria*, such as *Kocuria* and *Streptomyces*, which is consistent with their role in decomposing complex organic matter (94). Desert soils feature a high abundance of *Streptomyces*, a genus known for producing desiccation-resistant spores (95). Saline lakes are dominated by halotolerant genera, including *Halomonas* and *Marinobacter*, reflecting adaptations to osmotic stress (96).

Notably, our integrated approach, which combines enrichment culturing with metagenomics, recovered 4.02%–21.76% of the sequence-detected genera (Fig. 5d through f). This represents a 4- to 20-fold improvement over traditional cultivation methods. The enhancement was most pronounced in terrestrial habitats, where more moderate environmental conditions likely facilitate cultivability. In contrast, hypersaline lakes presented lower recovery rates (4.02%), remaining challenging to culture due to specialized nutritional or physiological requirements, such as high salt concentrations and low oxygen levels (66). These results underscore the persistent “great plate count anomaly” in extremely arid systems but demonstrate how this anomaly can be mitigated through tailored methodologies (97). The cultured collections, which span diverse functional groups, including nitrogen fixers, sulfate reducers, and halophiles, represent a valuable resource for exploring extremophile adaptations and biotechnological applications, such as enzyme discovery (98). However, the discrepancy between cultured and uncultured diversity highlights that the majority of microbial taxa in these habitats remain uncharacterized. These findings emphasize the continued need for optimized cultivation techniques to unlock the comprehensive genomic and functional potential of arid zone microbiomes.

### Methodological considerations and limitations

A limitation of this study is the use of different 16S rRNA gene regions for terrestrial and aquatic samples. The use of different primer sets for soils (V3-V4) and lake sediments (V4) may introduce amplification biases that affect alpha diversity comparisons. While our within-habitat analyses (e.g., salinity gradients) remain robust, cross-habitat diversity contrasts should be interpreted with caution, as primer differences could confound biological patterns. Our interpretations, therefore, emphasize within habitat patterns, including salinity-associated gradients within lake sediments and contrasts between wasteland and desert soils. All samples were clustered using closed reference operational taxonomic units against the SILVA 138 database to ensure a common phylogenetic framework for downstream analyses.

### Conclusions

This study elucidates how habitat heterogeneity, particularly salinity gradients and soil-lake distinctions, shapes microbial community assembly and function in the extremely arid Turpan–Hami Basin. Lake sediments presented significantly higher microbial diversity (OTU richness and Shannon index) than terrestrial wasteland and desert soils, with salinity inversely regulating diversity and driving taxonomic succession toward halophilic lineages such as *Halobacteria* in hypersaline lakes. Ecological assembly processes diverged across habitats: deterministic processes dominated desert soils and moderate-salinity lakes through heterogeneous/homogeneous selection, whereas stochastic processes dominated wastelands and extremely saline systems via dispersal limitation/drift. Metabolic functions were closely tied to habitat conditions; terrestrial ecosystems specialized in nitrogen cycling, while saline lakes exhibit salinity-partitioned sulfur metabolism, including prevalent sulfate respiration in high-salinity sediments. Co-occurrence networks revealed greater complexity in freshwater lakes than in extreme environments, reflecting contrasting resilience strategies. Cultivation efforts recovered 4.02%–21.76% of sequence-detected taxa, a 4- to 20-fold improvement over conventional methods though hypersaline lakes remained recalcitrant. These findings underscore the dual role of environmental filtering and dispersal limitation in arid ecosystems and highlight the need for integrated cultivation‒metagenomic approaches to bridge the “uncultured majority” gap and advance extremophile bioprospecting strategies.

## Data Availability

Sequence data that support the findings of this study have been deposited in the National Genomics Data Center with the primary accession code PRJCA044396 (wasteland) and the National Center for Biotechnology Information with the primary accession codes PRJNA1001621 (deserts) and PRJNA939974 (saline lakes).

## SUPPLEMENTAL MATERIAL

Supplemental material aem.02588-25-s0001.docx Tables S1 and S2; Fig. S1 to S4.

ASM does not own the copyrights to Supplemental Material that may be linked to, or accessed through, an article. The authors have granted ASM a non-exclusive, world-wide license to publish the Supplemental Material files. Please contact the corresponding author directly for reuse.
