# Supplementary material for: Habitat heterogeneity drives microbial community assembly and functional specialization in extremely arid ecosystems
Source: Appl Environ Microbiol. 2026 Jan 27;92(2):e02588-25. doi: 10.1128/aem.02588-25 (PMC12915352; doi:10.1128/aem.02588-25)
Supplement: Supplemental material — Tables S1 and S2; Fig. S1 to S4. [file aem.02588-25-s0001.docx]

**Supplementary materials for**

**Habitat heterogeneity** **drives microbial community assembly and functional specialization in extremely arid ecosystems**

Jianrong Huang^1^**^**, Min Cai^2^**^**, Mingxian Han^2^, Baozhu Fang^3^, Lei Dong^4^, Gaosen Zhang^5^, Jia-Rui Han^4^, Shuai Li^3^, Nigora Rustamova^6^, Yonghong Liu^3^*, Wen-Jun Li^4^, Hongchen Jiang^1,2,3^*

^1^School of Life Sciences, Henan University, Kaifeng 475001, China.

^2^State Key Laboratory of Geomicrobiology and Environmental Changes, China University of Geosciences, Wuhan 430074, China.

^3^State Key Laboratory of Ecological Safety and Sustainable Development in Arid Lands, Xinjiang Institute of Ecology and Geography, Chinese Academy of Sciences, Urumqi, 830011, China.

^4^School of Life Sciences, Sun Yat‑sen University, Guangzhou 510275, China.

^5^Key Laboratory of Extreme Environmental Microbial Resources and Engineering, Northwest Institute of Eco-Environment and Resources, Chinese Academy of Sciences, Lanzhou 730000, China.

^6^Institute of Microbiology, Academy of Sciences of the Republic of Uzbekistan, Tashkent, Shaykhantakhur district, street Abdulla Kadiriy, 7 B100128, Uzbekistan.

*Correspondence:

Hongchen Jiang, Jiangh@henu.edu.cn

Yonghong Liu, liuyh@ms.xjb.ac.cn

**^**Equal contribution.

**Table S1** Media employed for the cultivation work in the Turpan-Hami Basin samples.

| Media | Habitat | References |
| --- | --- | --- |
| TRYPTONE SOYA BROTH (TSB) | Wastelands | DSMZ_Medium545 |
| Gauze's Synthetic Medium No.1 | Wastelands | DSMZ_Medium1048 |
| YMA | Wastelands | DSMZ_Medium1031 |
| ISP 2 | Wastelands | DSMZ_Medium987 |
| ISP 5 | Wastelands | DSMZ_Medium993 |
| Marine medium | Wastelands | DSMZ_Medium1303 |
| King's B | Deserts, saline lakes | ^[1]^ |
| Marine Agar 2216 | Deserts, saline lakes | DSMZ_Medium604 |
| R2A medium | Deserts, saline lakes | ^[2]^ |
| Nutrient Agar | Saline lakes | DSMZ_Medium1 |

Note: All media should be supplemented with 10 mL of trace salt solution (per 100 mL). DSMZ medium can be accessed through the website (<https://www.dsmz.de>).

**Table S2** Media recipe of trace salts, inhibitor combination and vitamin B complex solution.

| Additives | Recipe | Details |
| --- | --- | --- |
| Trace salts | **(per 100 mL)** Ferrous sulfate heptahydrate 0.1 g, manganese sulfate 0.1 g, zinc sulfate 0.1 g | Dissolve thoroughly in sterilized deionized water, filter-sterilize, then seal and store in a 4°C refrigerator for later use. Storage period should not exceed one month. |
| Inhibitor combination | **(per 1000 mL)** 1 mL of 25 mg/mL nalidixic acid, 1 mL of 25 mg/mL nystatin, and 1 mL of 50 mg/mL cycloheximide. | All three inhibitors were filter-sterilized using a 0.22 μm filter membrane. |
| Vitamin B complex solution | **(per 100 mL)** 50 mg inositol, 50 mg thiamine, 50 mg riboflavin, 50 mg niacin, 50 mg calcium pantothenate, 50 mg pyridoxine hydrochloride, 50 mg p-aminobenzoic acid, 25 mg biotin, 25 mg cobalamin, and 25 mg tetrahydrofolate. | After thorough stirring and homogenization, the solution was filter-sterilized with a 0.22 μm filter membrane, and 1 mL was added per liter of medium. |

**Fig. S1** Geospatial distribution of microbial sampling sites across the heterogeneous habitats of the Turpan-Hami Basin: The depicted sites encompass a broad spectrum, including Wastelands (BSS, n=77), Deserts (DES, n=72), and Saline Lakes (SAL, n=117).


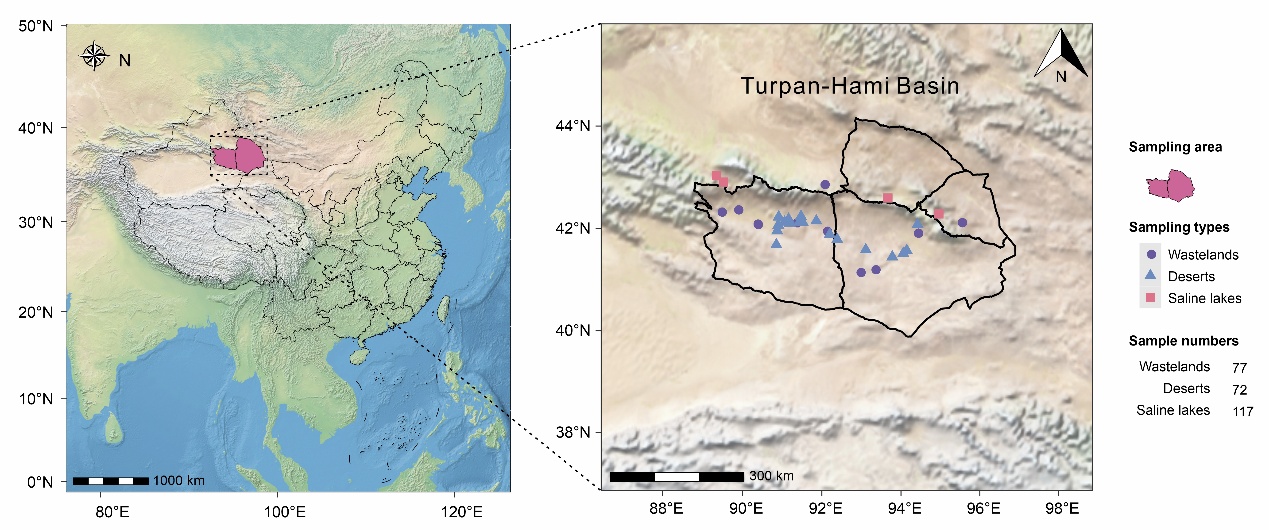


**Fig. S2** Distance-based redundancy analysis (dbRDA) showing the influence of environmental factors on microbial community structures. (a) Correlations between environmental variables and microbial communities in the SAL habitat. (b) Relationships between environmental variables and microbial communities across different treatments within the SAL habitat. The percentages on the axes represent the proportion of variation explained by each coordinate.


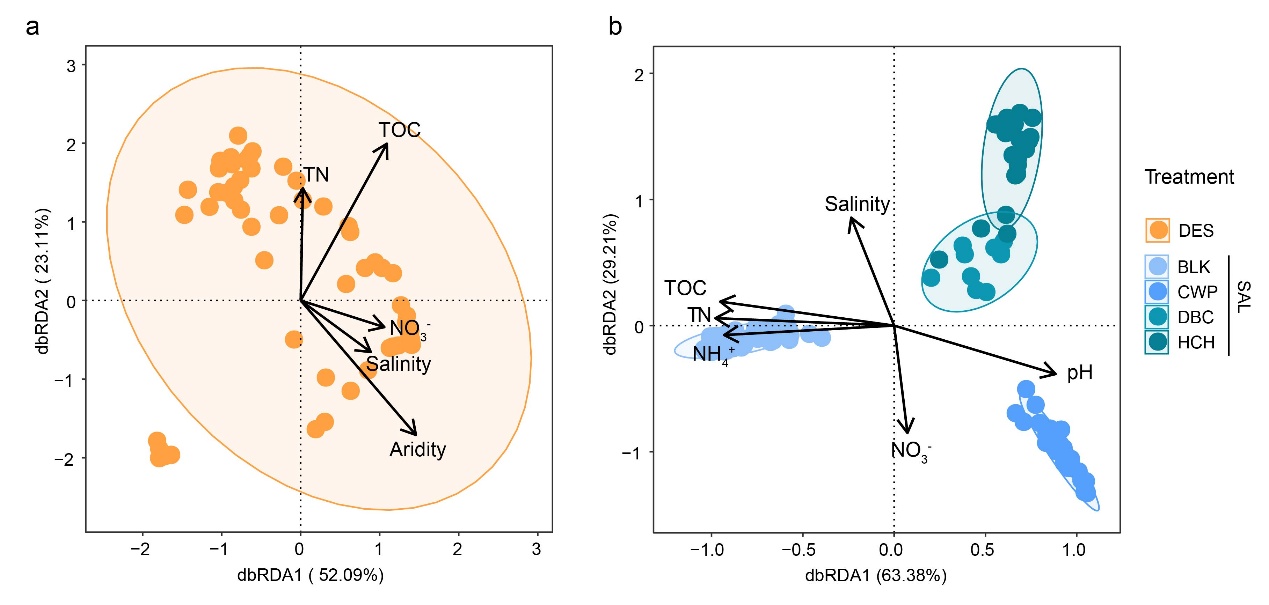


**Fig. S3.** Topological roles of microbial taxa in co-occurrence networks across different habitats based on Zi-Pi plots. Colored labels beside the plots denote the taxonomic identity of identified keystone taxa (module hubs, connectors, and network hubs) at the class level.


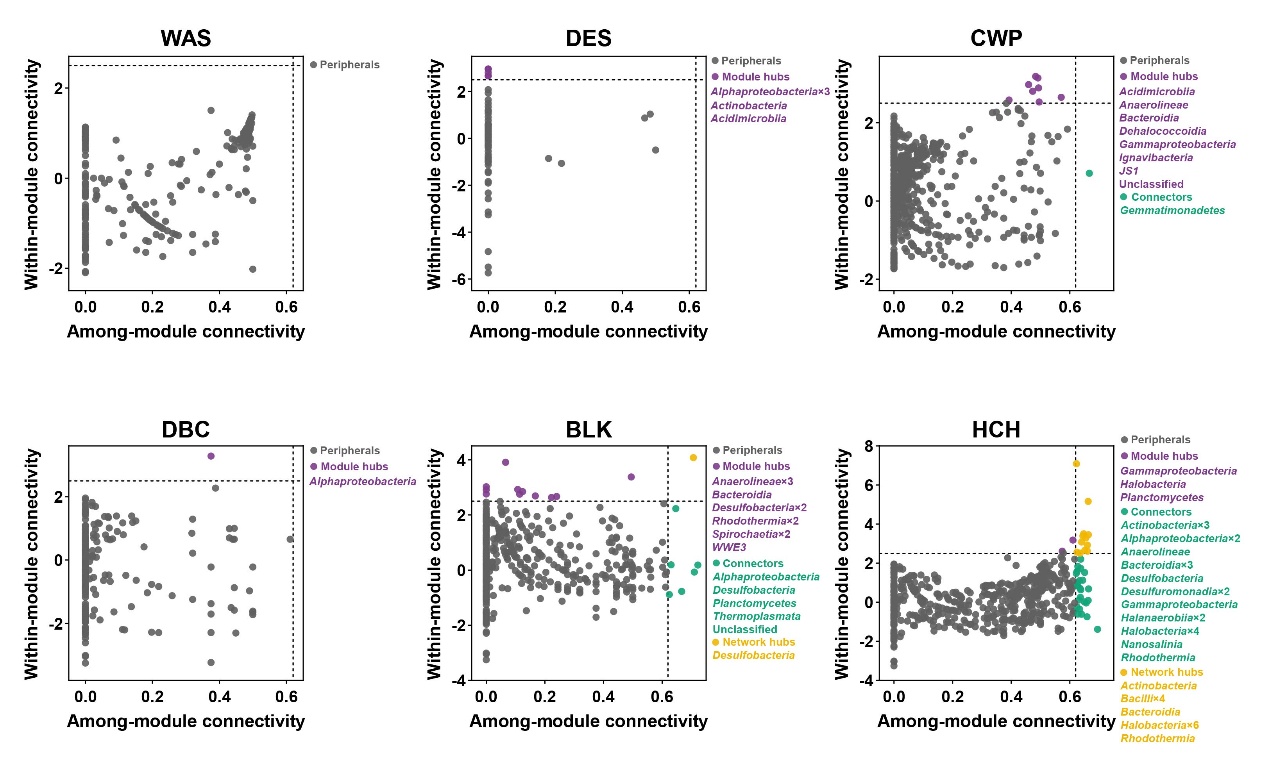


**Fig. S4** Predicted functional profiles of culturable microbial communities across three habitats (WAS: wastelands; DES: deserts; SAL: saline lakes) via FAPROTAX. (a) Habitat-specific microbial functional profiles, where circle size corresponds to the relative abundance of predicted functions. (b) Bar plots quantifying the cumulative relative abundance of carbon (C), nitrogen (N), and sulfur (S) metabolism across the three habitats.

**
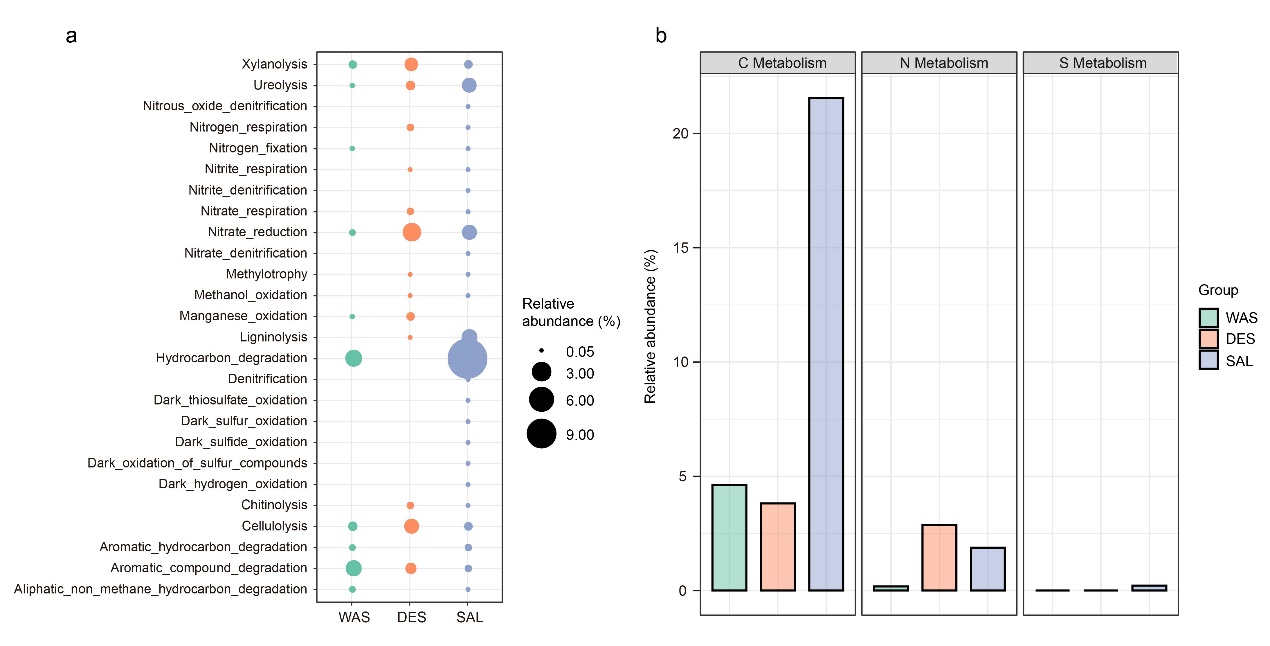
**

**References**

1. King EO, Ward MK, Raney DE. Two simple media for the demonstration of pyocyanin and fluorescin. **The Journal of Laboratory and Clinical Medicine** **1954**, 44(2)**:** 301-307.

2. Reasoner DJ, Geldreich EE. A new medium for the enumeration and subculture of bacteria from potable water. **Applied and Environmental Microbiology** **1985**, 49(1)**:** 1-7.
